# Lipid Nanoparticle-Mediated Liver-Specific Gene Therapy for Hemophilia B

**DOI:** 10.3390/pharmaceutics16111427

**Published:** 2024-11-09

**Authors:** Brijesh Lohchania, Porkizhi Arjunan, Gokulnath Mahalingam, Abinaya Dandapani, Pankaj Taneja, Srujan Marepally

**Affiliations:** 1 Department of Biotechnology, School of Engineering and Technology, Sharda University, Greater Noida 201310, Uttar Pradesh, India; 2021387533.brijesh@dr.sharda.ac.in; 2 Centre for Stem Cell Research (CSCR) (a Unit of inStem, Bengaluru), Christian Medical College Campus, Vellore 632002, Tamil Nadu, India

**Keywords:** non-viral delivery, FIX-L (Padua mutant), hemophilia B, lipid nanoparticles, gene therapy

## Abstract

**Background**/**Objectives**: Hemophilia B is a hereditary bleeding disorder due to the production of liver malfunctional factor IX (FIX). Gene therapy with viral vectors offers a cure. However, applications are limited due to pre-existing antibodies, eligibility for children under 12 years of age, hepatotoxicity, and excessive costs. Lipid nanoparticles are a potential alternative owing to their biocompatibility, scalability, and non-immunogenicity. However, their therapeutic applications are still elusive due to the poor transfection efficiencies in delivering plasmid DNA into primary cells and target organs in vivo. To develop efficient liver-targeted lipid nanoparticles, we explored galactosylated lipids to target asialoglycoprotein receptors (ASGPRs) abundantly expressed on hepatocytes. **Methods**: We developed 12 novel liposomal formulations varying the galactose lipid Gal-LNC 5, cationic lipid MeOH16, DOPE, and cholesterol. We evaluated their physicochemical properties, toxicity profiles, and transfection efficiencies in hepatic cell lines. Among the formulations, Gal-LNC 5 could efficiently transfect the reporter plasmid eGFP in hepatic cell lines and specifically distribute into the liver in vivo. Toward developing functional factor IX, we cloned Padua mutant FIX-L in a CpG-free backbone to enhance the expression and duration. **Results**: We demonstrated superior expression of FIX with our galactosylated lipid nanoparticle system. **Conclusions**: The current research presents a specialized lipid nanoparticle system viz. Gal-LNC which is a specialized lipid nanoparticle system for liver-targeted gene therapy in hemophilia B patients that has potential for clinical use. The Gal-LNC successfully delivers a CpG-free Padua FIX gene to liver cells, producing therapeutically relevant levels of FIX protein. Among its benefits are the ideal qualities of stability, targeting the liver specifically, and maximizing efficiency of transfection. Optimization of liver-targeting lipid nanoparticle systems and function FIX plasmids will pave the way for novel lipid nanoparticle-based gene therapy products for hemophilia B and other monogenic liver disorders.

## 1. Introduction

Hemophilia B is an X-linked bleeding disorder characterized by a deficiency in human coagulation factor IX, impacting approximately 1 in 25,000 individuals in the general population. This condition can lead to internal bleeding in joints and muscles, resulting in significant joint pain, deformities, and a loss of mobility [1,2,3]. The current treatment landscape for hemophilia B involves the repeated intravenous administration of clotting factor concentrates (CFC) or recombinant factor IX (rFIX). Due to the shorter half-life of these treatments, patients typically require two to three intravenous injections each week to effectively prevent hemorrhages and mitigate secondary complications, such as hemophilic arthropathy [4]. However, the necessity for frequent injections poses several challenges. Patients are at an increased risk of developing infections from repeated venous access, and the inconvenience of regular treatments can be burdensome [5]. Moreover, approximately 30% of pediatric patients may develop inhibitors to FIX, complicating their treatment further. This situation necessitates bypassing agents for hemostasis and immune tolerance induction, both complex and costly [6]. These treatment limitations contribute to a significant disparity in care, particularly in developing countries, where nearly 70–80% of hemophilia patients lack access to adequate treatment options. This gap highlights the urgent need for improved therapeutic solutions and greater accessibility to care for individuals affected by this condition [7,8]. Due to these limitations of current protein replacement therapies, gene therapy has emerged as a promising alternative for hemophilia B patients. A single injection of functional protein-encoding pDNA could provide long-term protein expression, eliminating the need for repeated protein injections [9,10]. Gene therapy can remarkably improve the disease phenotype to a mild state with as little as 5% of endogenous clotting factor levels. This minimal expression can effectively eliminate the risk of spontaneous bleeding events and negate the necessity for prophylactic protein therapy, significantly enhancing the quality of life for patients [11].

Nathwani AC et al. demonstrated that a single dose of adeno-associated virus (AAV) carrying the FIX gene could reduce bleeding episodes by 90% in six hemophilic patients without inducing toxicity or immunological issues throughout a year-long observation period [12]. Further, they demonstrated that delivering a Padua variant of the FIX gene (R338L) using AAV resulted in sustained therapeutic expression of FIX coagulant activity after gene transfer in ten participants with hemophilia who received the same vector dose [13]. Despite these successful clinical trials, AAV-based gene therapy could not reach a broader disease population due to low patient eligibility, particularly regarding pre-existing antibodies [14,15,16,17,18]. The administration of high-titer AAV can lead to systemic toxicity and immune provocations [19,20]. Exorbitant costs such as hemophilia B gene therapy product, Hemgenix, cost 3.5 mn USD per dose [21].

Recent advances in lipid nanocarrier-based nucleic acid therapies showed encouraging results in clinical research. Alton EW et al. demonstrated the efficacy of cationic lipid-based vectors in delivering the larger Cystic Fibrosis Transmembrane Conductance Regulator (CFTR) gene (189 Kb) during phase 2 clinical trials, resulting in a partial restoration of the ion channel function in patients suffering from CFTR-related diseases [22]. In a parallel development, Alnylam Pharmaceuticals received FDA approval for ONPATTRO™, a lipid-based RNA interference (RNAi) drug for amyloidosis [23]. These promising outcomes inspire us to develop safer, more effective therapeutic options with lipid nanoparticles for treating hemophilia B.

Liver cells, particularly hepatocytes, are crucial in producing blood clotting factors essential for maintaining hemostasis. A promising therapeutic strategy involves targeting asialoglycoprotein receptors (ASGPRs), which are abundantly expressed on the surface of hepatocytes. This approach has garnered significant attention due to its potential efficacy [24]. Galactose is a potent ligand for ASGPRs, enhancing the targeting capabilities of therapeutic agents [25]. Notably, synthetic galactosylated analogues have demonstrated a marked increase in binding affinity to ASGPRs [26]. These galactosylated lipids represent a promising avenue for delivering therapeutic genes directly into hepatocytes, particularly for treating liver disorders [27]. Our prior findings demonstrated that cationic lipids modified with β-D-Galactose ligands exhibit enhanced ASGPR targeting properties and efficiently deliver reporter genes to the mouse liver [28]. Taking cues from our previous findings, we developed a lipid nanoparticle system utilizing the natural ligand β-D-Galactose, anticipating that it will provide a safer and more effective strategy for liver-specific gene delivery. We chose CpG-free human factor IX of the Padua variant to develop gene therapy for hemophilia B. The Padua variant of factor IX, having an amino acid change at R338L, demonstrated a gain of functions in clotting activity over 8-fold compared to the wild type [13]. More importantly, it is a naturally occurring mutation, and the safety of the variant is also well established. Hence, this hypervariant showed significance in advancing gene therapy for hemophilia B [29]. Prior findings demonstrated that CpG-free plasmids extended the duration of transgene expression in vivo. The removal of CpG dinucleotides enhances the degree and duration of transgene expression, especially in organs like the liver and spleen, and maintains the expression of the delivered genes at a therapeutic level over extended periods [30]. This makes it relevant for therapeutic applications in treating inherited diseases such as cystic fibrosis and hemophilia. Building on these insights, we developed a novel galactosylated nanoparticle system designed to effectively deliver CpG-free Padua FIX plasmid DNA (pDNA) to hepatic cell lines, thereby facilitating the production of functional FIX protein.

## 2. Materials and Methods

### 2.1. Materials

1,2-dioleoyl-sn-glycero-3-phosphoethanolamine (DOPE) and cholesterol were purchased from Avanti Polar (Alabaster, AL, USA). ZetaSizer, from Malvern Panalytical (Malvern, UK). The ChemiDoc MP Imaging system was used for all gel imaging. Dulbecco’s Modified Eagle Medium (DMEM) from MP Biomedicals (Santa Ana, CA, USA) and Eagle’s Minimum Essential Medium (EMEM) from Himedia (Kelton, PA, USA) were used for cell line culture. SpectraMAx i3X from Molecular Devices (San Hose, CA, USA) for cytotoxicity absorbance reading. BD FACS Celesta analyzed the flow cytometry (Franklin Lakes, NJ, USA), and eGFP fluorescence imaging was done using a Leica DMi8 Fluorescence Microscope (Wetzlar, Germany). All the flow cytometric data was analyzed by FlowJo Software-10.10.0 Version. Christ Alpha 1-2Plus (Osterode am Harz, Germany) was used for lyophilization. qPCRs were performed using QuantStudio Real-Time PCR Systems from Applied Biosystems, Thermo Fisher Scientific (Waltham, MA, USA). Mice from Jackson Laboratory (Bar Harbor, ME, USA) are maintained under the regulations of the CSCR animal facility—BALB/c mice (male, 8 weeks old). Using the Living Image, Caliper Lifesciences, a PerkinElmer company (Hopkinton, MA, USA), estimated the ROI measurement^®^ of Software 4.4 for the IVIS spectrum.

### 2.2. Methods

#### 2.2.1. Lipid Synthesis

The 16 Cyclic-6-Gal lipid (16 Cyclic-6-Gal lipid was referred to as lipid 3 in the article) [28,31] was synthesized using previously established protocols.

#### 2.2.2. Galactosylated Lipid Nanocarrier (Gal-LNC) Preparation

For galactosylated liposomes, varying molar ratios of MeOH 16, Cholesterol, and DOPE were dissolved in a chloroform-methanol mixture (3:1) in a glass vial (Table 1), keeping galactosylated lipid constant. The organic solvent mixture was evaporated under nitrogen gas to form a thin lipid film and subjected to vacuum drying for 2 h. Next, 1 milliliter of sterile deionized water was added to the thin film and hydrated overnight. Next, it is subjected to vortexing for two minutes at room temperature to form multi-lamellar vesicles (MLVs). Subsequently, the MLVs were sonicated for 5 min in a water bath and 1 min for 15 s pulse and 15 s intervals in a probe sonicator to form small uni-lamellar vesicles, or SUVs.

#### 2.2.3. Characterization of Liposomes by Dynamic Light SCATTERING (DLS) Technique

Using Zetasizer equipment, liposomes were evaluated for their hydrodynamic diameter (HDD), Zeta potentials, and polydispersity index (PDI) in Milli ‘Q’ water. Liposomes were inserted into folded capillary cells for measurement. Three readings were taken of every measurement.

#### 2.2.4. pDNA Gel Retardation

The electrostatic binding interactions between cationic liposomes and nucleic acids (pDNA) were investigated in an agarose gel retardation assay. Lipoplexes (liposomes with pDNA) were made at a varying N/P ratio (nitrogen to phosphate) from 1:1 to 8:1 and allowed to be incubated for 20 min at room temperature. The lipoplexes containing 6× loading dye were loaded into 1% agarose gel; pDNA alone was used as a control.

#### 2.2.5. Heparin Displacement

Lipoplex was prepared using a method similar to the one described above. Following, 10 µL of negatively charged heparin of 1 mg/mL concentration was added to the lipoplex, followed by a 20-min incubation period. The resulting mixtures were subsequently analyzed through 1% agarose gel electrophoresis.

#### 2.2.6. DNase Sensitivity Assay

5 µL of 1× DNase buffer and 0.5 units of DNase I were added to the lipoplex, followed by a 30-min incubation. Subsequently, 5 µg of proteinase K was added, and the mixture was maintained at 50 °C for 15 min. Afterward, 100 µL of a 1:1 phenol–chloroform solution was added and incubated at room temperature for 5 min. This was followed by centrifugation at 14,000 RPM in 15 min. Finally, DNA loading dye was added to the aqueous layer, and the results were analyzed using 1% agarose gel electrophoresis.

#### 2.2.7. In Vitro Cytotoxicity

The cytotoxicity of the liposomes was assessed utilizing the MTT reagent-based assay. A total of 10,000 HEK293T cells were initially plated in 96-well plates and incubated at 37 °C in a 5% CO_2_ atmosphere for approximately 16–20 h to reach 70−80% confluence. Following this, the lipoplex was transfected, and the cells were incubated again at 37 °C in 5% CO_2_ for 48 h. Subsequently, 20 μL of MTT (5 mg/mL in PBS, pH 7.4) was added and incubated for 4 h. After this incubation, the medium was carefully removed, and 150 μL of DMSO was added to dissolve the resulting formazan crystals. The absorbance was then measured at 570 nm using a microplate reader (Spectra Max i3X).

#### 2.2.8. Uptake Assay

For cellular localization experiments, 1% Rho-PE was used in preparing the red fluorescent liposomes, maintaining the concerned lipid ratios (Table 1), followed by transfection, and the uptake % was calculated by flow cytometry after 4 h of post-transfection.

#### 2.2.9. pDNA Transfection

Transfection experiments were carried out using HEK293T and HepG2, two distinct cell lines. Cells were plated at a density of 45,000 cells per well in a 48-well plate and incubated for 16 h before transfection. The lipoplex was formulated with LNC and Gal-LNC at pDNA-to-lipid N/P charge ratios of 1:1 in a serum-free medium, followed by a 30-min incubation at room temperature. A total of 0.4 µg of eGFP pDNA, which encodes the plasmid’s green fluorescent protein (GFP) size at 4.7 kb, was used in this study. After the incubation period, the lipoplex was transfected to the cells, which were maintained at 37 °C in a 5% CO2 environment. GFP expression was evaluated 48 h later by visualizing the cells under a fluorescent microscope (Leica DM100) and through FACS analysis using a BD Celesta.

#### 2.2.10. FIX Plasmid Construct

The codon-optimized factor IX with Padua-L mutant gene block was designed and purchased, and the gene block was inserted into the Kpn1- and Nhe1-digested backbones of CpG-free plasmid by Gibson assembly. Sequencing PCR was used to confirm the size of the cloned plasmid by using a primer set (Table S1), and restriction digestion was also utilized to confirm the FIX-L plasmid cloned construct again. The plasmid was expanded by the transformation in the presence of Zeocin, and the plasmid was isolated using low-salt LB agar. Snapgene software v. 5.1 was used to design this construct.

#### 2.2.11. Western Blot Analysis

Since factor IX (FIX) is a secreted protein, the media supernatants were collected from the respective samples, and the concentrations were quantified using the Bradford assay with BSA standards. A total of 50 μg of protein lysates was resolved on a 10% SDS-PAGE gel and subsequently transferred to a PVDF membrane (WH3135834). Following a blocking step with 5% bovine serum albumin in TBST, the membrane was incubated with a human factor IX antibody (Thermo Scientific, MA5-29254, diluted 1:1000 in blocking buffer). This was followed by incubation with goat anti-rabbit IgG H&L-HRP (Invitrogen, Walhtam, MA, USA, ab205718, diluted 1:10,000 in blocking buffer). The presence of human factor IX was visualized using a chemiluminescence substrate on the Chem-Doc imaging system (Bio-Rad, Hercules, CA, USA).

#### 2.2.12. Quantitative Real-Time Reverse Transcription-PCR (qRT-PCR)

We used the protocol for qRT-PCR as described. Total RNA was isolated from one million cells using RNA iso-plus reagent per manufacture protocol (Takara, Kusatsu, Japan) from HEK293T and HepG2 cells. A total of 500 ng of total RNAs were converted into cDNA using a cDNA synthesis kit (Takara). SYBER^®^ Green I-based qPCR was performed using primer sets (Table S2) with 50 ng of cDNA from each sample in the QuanStudio-6 qPCR system (Applied Biosystems) with thermocycler condition: Hold Stage: 95 °C for 10 min; PCR Stage: 40 cycles of 95 °C for 10 s and 60 °C for 30 s; Melt Curve Stage: 95 °C for 15 s and 60 °C for 1 min, and 95 °C for 15 s in the real-time PCR system. The Ct value of human factor IX normalized with the Ct value of internal control (GAPDH gene expression) in each sample, and fold change was calculated by the 2^− ΔΔCT^ method.

#### 2.2.13. Lyophilization of Liposome

A total of 25 µg of plasmid DNA was dissolved in a 5% sucrose solution with a total volume of 125 µL, and a 1:1 ratio of liposome was calculated for 25 µg of pDNA that was also dissolved in a 5% sucrose solution with a total volume of 125 µL. Add both solutions together and incubate for 30 min at room temperature. The lipoplex was frozen in liquid nitrogen for 15 min and stored at −80 °C for 2 h. Then, the samples were lyophilized for 16 h (Christ Alpha 1-2Plus).

#### 2.2.14. In Vivo Imaging

NIR dye (DiLC18, (1,1′-Dioctadecyl-3,3,3′,3′-Tetramethylindocarbocyanine Perchlorate, Thermo Scientific) labeled lipoplexes were prepared similarly and subjected to lyophilization. For each mouse (n = 3), 100 µL lipoplex volume was administered intraperitoneally into BALB/c mice (i.p). After 24 h of injection, epifluorescence was measured using an in vivo imaging system (IVIS).

#### 2.2.15. Statistical Analysis

Ordinary one-way ANOVA and multiple t-tests were used in the statistical analysis, and they were done with GraphPad Prism (Version 8.02, GraphPad Prism).

## 3. Results

### 3.1. Characterization of LNC and Gal-LNC

**Figure 1:**
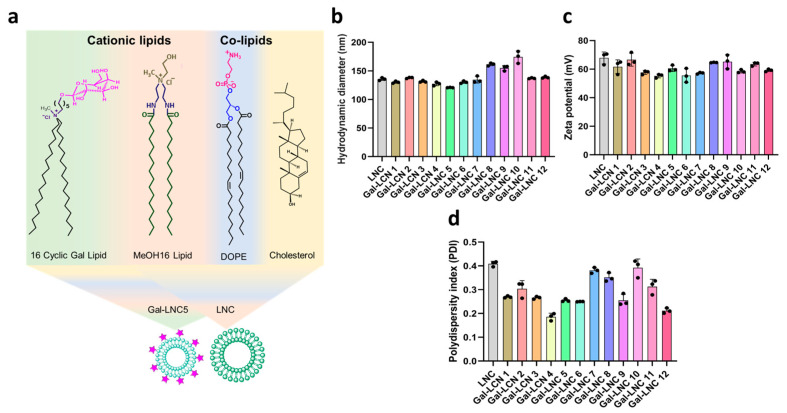
LNC and Gal-LNC characterizations. (**a**) Lipid structures of LNC and Gal-LNC, (**b**) size, (**c**) zeta, and (**d**) polydispersity indexes of LNC and Gal-LNC (n = 3).

The features of the Gal-LNC, such as their size falling within the nm range, uniform dispersion in solution, and positive charge of less than, make them promising candidates for non-viral gene therapy. Various combinations of liposomes were prepared by utilizing different concentrations of lipids and co-lipids, as detailed in Table 1 with their concentrations used. The structure of lipids, co-lipids, and the formation of liposomes are illustrated in Figure 1a. As presented in Figure 1b, the size of the Gal-LNC falls within the 200 nm range. The ζ potentials confirm successful quantification, reassuring our measurements’ accuracy, with all liposomes demonstrating a positive charge of less than +70 mV (Figure 1c). Additionally, all Gal-LNC exhibit uniform dispersion in solution, with a polydispersity index (PDI) value below 0.5, as shown in Figure 1d.

### 3.2. Liposomes Complexation Study of pDNA

**Figure 2:**
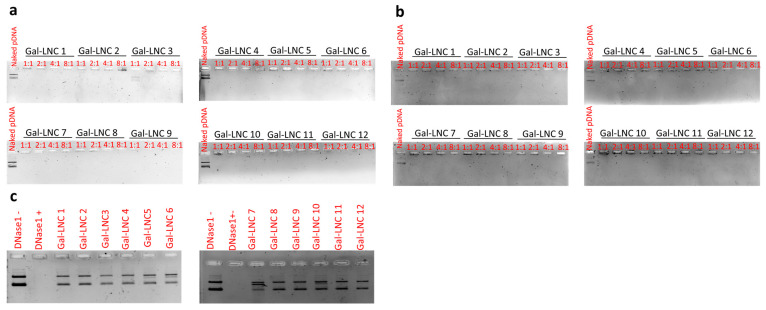
Liposomes complexation study of pDNA. (**a**) Electrophoretic mobility retardation assay of naked pDNA, LNC, and Gal-LNC with charge ratios (1:1, 2:1, 4:1, and 8:1); (**b**) Heparin displacement assay of naked pDNA, LNC and Gal-LNC lipoplex in the presence of heparin; (**c**) DNase Sensitivity assay for LNC and Gal-LNC in the presence of DNase I enzyme.

To evaluate the binding efficacy of liposomes, a gel retardation assay was conducted, yielding detailed results that demonstrate robust complexation of all Gal-LNC variants (1–12) with pDNA across all tested N/P ratios, including 1:1, 2:1, 4:1, and 8:1, as illustrated in the accompanying Figure 2a. Additionally, a heparin displacement assay assessed the competitive interactions between nucleic acids and other negatively charged molecules in the bloodstream. Heparin, a highly negatively charged polysaccharide integral to the extracellular matrix of various tissues and found on cell surfaces, was introduced to the lipoplex containing pDNA. Remarkably, the presence of heparin did not compromise the stability of the lipoplex against competitive binding, as shown in Figure 2b. Furthermore, a DNase I protection assay confirmed the liposomes’ capability to protect complexed pDNA (1:1 N/P charge ratio) from endonuclease degradation. Following the introduction of DNase I and proteinase K to the lipoplex, along with phenol: chloroform, unprotected pDNA exhibited no degradation, resulting in the detection of a band, which serves as the negative control (Figure 2c). In contrast, adding DNase I to naked pDNA led to hydrolysis of the pDNA backbone, affirming its liability. In absolute comparison, the lipoplexes successfully evaded degradation and accumulated in the aqueous phase after the phenol–chloroform treatment.

### 3.3. In Vitro Compatibility of LNC and Gal-LNC

**Figure 3:**
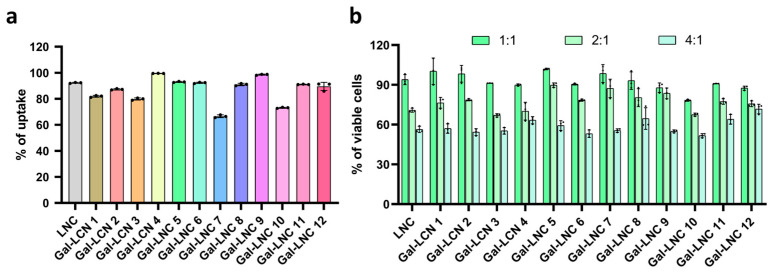
In vitro compatibility of LNPs. (**a**) Uptake assay of LNC and Gal-LNC 5, % of uptake analyzed by Flow cytometry, (**b**) Cytotoxicity assay of LNC and Gal-LNC (n = 3).

Before assessing transfection efficiency, we confirmed the primary principle of cellular uptake for the liposomes. The cellular uptake assay for LNC and Gal-LNC revealed a notable 90% uptake after just 4 h when we tested with a 1:1 N/P charge ratio, as illustrated in Figure 3a. To further validate the safety of our nanoparticles, we evaluated the cytotoxicity of both LNC and Gal-LNC using the MTT assay with HEK293T cells. At a 1:1 N/P charge ratio, both formulations exhibited over 95%, even a few liposomes at 100% cell viability. However, at a 2:1 N/P charge ratio, cell viability decreased to between 70% and 80% and was further reduced to below 60% at higher ratios (Figure 3b).

### 3.4. Screening of Liposome Efficiency by Transfecting pDNA

**Figure 4:**
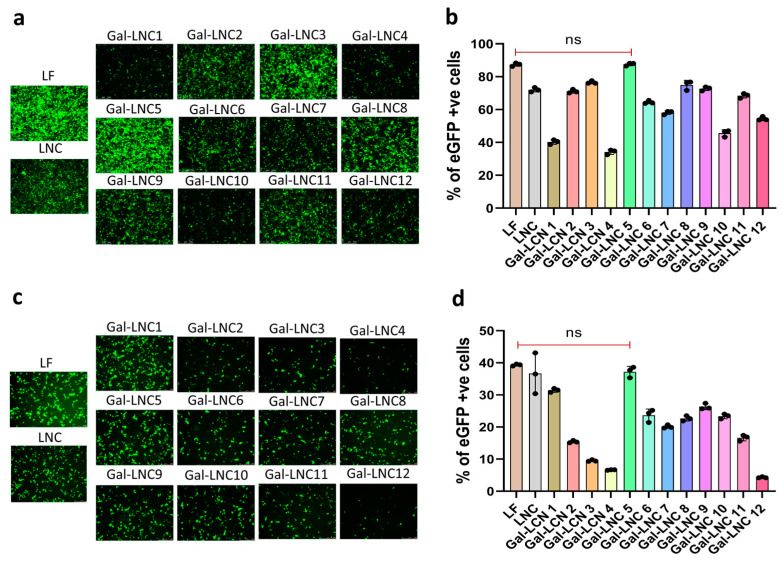
Screening of liposome efficiency by transfecting pDNA. (**a**) Transfections of LNC and Gal-LNC screening in HEK293T with pDNA, the qualitative analysis by imaging; (**b**) Quantitative analysis by flow cytometry (n = 3), (**c**) Transfections of LNC and Gal-LNC screening in HepG2 with pDNA, the qualitative analysis by imaging; (**d**) Quantitative analysis by flow cytometry % of eGFP cells represents the overall percentage of positive cells.

Galactosylated liposomes were evaluated for their transfection capabilities in Hek 293T and HepG2 cell lines, with performance compared to the commercial control, Lipofectamine p3000 (LF), as shown in Figure 4a. Both flow cytometry and imaging methods provided robust statistical and qualitative validation of transfection efficacy (Figure 4a,b). Table S3 shows the statistical significance and p values; the 5th is nonsignificant with LF, whereas all Gal-LNC were significantly lower (Table S4). The fifth composition emerged as the most effective among the various formulations tested from the liposome library. Since hemophilia is a liver disease, we specifically targeted hepatic cell lines to assess the transfection efficiency of these liposomes. In vitro transfections using Gal-LNC in HepG2 cells with eGFP pDNA demonstrated transfection efficiency comparable to that of the LF at a 1:1 charge ratio of liposome to pDNA. As anticipated, the liver-targeted liposomes exhibited efficiency on par with the commercial counterpart, as illustrated in Figure 4c,d. The statistical significance and p values; the fifth is non-significant with LF, though all Gal-LNC were notably less (Table S4). Finally, after selecting the best liposomes, we comprehensively compared the complexation and sensitivity assays between LNC and Gal-LNC by varying the charge ratios of 1:1, 2:1, 4:1, and 8:1 (Figure S1a–c). This comparison exposed the potential of Gal-LNC 5 for efficient complexation and stability for transfections.

### 3.5. FIX-L Expression by Gal-LNC 5

**Figure 5:**
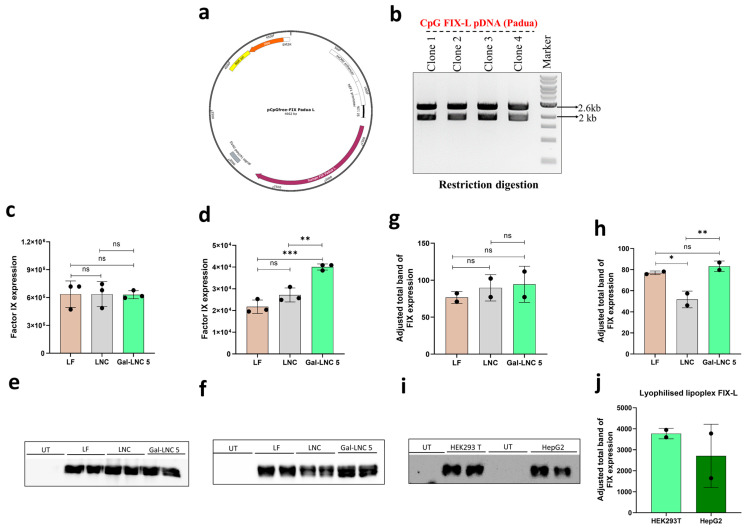
FIX-L pDNA construct and expression. (**a**) FIX-L gene construct in CpG-free sequence backbone; (**b**) The clone was confirmed by sequencing and restriction digestion (n = 4); (**c**) The plasmid expression in non-hepatic cell line HEK293T by qPCR (n = 2); (**d**) The plasmid expression in non-hepatic cell line HepG2 by qPCR (n = 2) (**e**) The FIX-L pDNA protein expression was confirmed by Western blot in HEK293T cells; (**f**) The FIX-L pDNA protein expression was confirmed by Western blot in HepG2 cells; (**g**) The bands were quantified by ImageJ software v. 3.0.1.14—HEK293T (refer c) (n = 2) (**h**) The bands were quantified by ImageJ software—HepG2 (refer f) (n = 2) (**i**) Western blot analysis for FIX-L pDNA in HEK293T cells expression of media supernatant (**j**) The bands were quantified by ImageJ software (n = 2). ns—non-significant; * *p* < 0.05; ** *p* < 0.01; *** *p* < 0.001. UT—untreated; LF—Lipofectamine 3000.

Subsequently, the CpG-free FIX-L plasmid was prepared. We employed restriction digestion and sequencing techniques to confirm the successful cloning of the FIX-L plasmid, as illustrated in Figure 4a,b. The functional analysis of the CpG-free FIX-L pDNA was quantified using qPCR in both HEK293T and HepG2 cell lines (Figure 4c). The results displayed that the FIX expression was non-significant between the groups; the Gal-LNC 5 is equivalent to LF with the expression range of 7 × 10^5^; on the other side, the HepG2 hepatic cell line shows an attractive result that LF gives lesser expression than both our LNC and Gal-LNC 5-LNC group; this may be because HepG2 was hard to transfect cell line. The Gal-LNC 5 reached 4 × 10^4^, which is more significant than LF 2 × 10^4^. This shows that the Gal-LNC 5 is more efficient in transfecting cells that are challenging to transfect (Figure 5d). For protein expression, Western blot was used, and the same qPCR results were replicated in WB; briefly, the non-hepatic cell line HEK293T was equivalent in all the groups, including LF, LNC, and Gal-LNC 5 (Figure 5e,g). However, HepG2 Gal-LNC 5 had more significant protein expressions than LNC and was comparable to LF (Figure 5f,h).

Western blot analysis was conducted further to investigate the stability of the Gal-LNC 5 post-lipoplex formation; the complex was frozen, lyophilized, and stored at 4 °C. After a week, it was reconstituted with serum-free media and transfected into HEK293T and HepG2 cells. The Western blot results demonstrated that the lipoplex remained stable following lyophilization and effectively expressed the therapeutic protein factor IX-L without compromising the integrity of the gene. The diluted state of the lipoplex facilitates better control over electrostatic interactions between the liposomes and nucleic acids. Notably, lyophilized products do not harm the target gene during extended storage, allowing for the versatile distribution of distinct liposomes. This formulation has the potential to provide an improved clinical product while ensuring the long-term stability of the drug (Figure 5i,j).

### 3.6. Biodistribution of Gal-LNC 5

**Figure 6:**
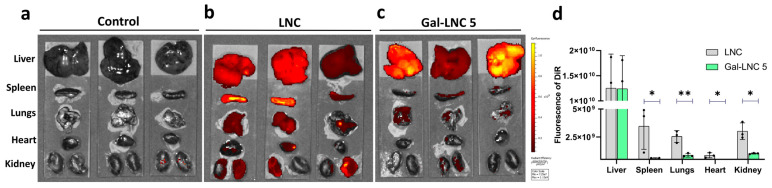
Biodistribution of 16 cyGAL5. (**a**) Control mice; (**b**) Localization of LNC to the liver and other organs; (**c**) Uptake of Gal-LNC 5 to liver; (**d**) Epifluorescence of Gal-LNC 5 (n = 3) * *p* < 0.01 ** *p* < 0.001.

The biodistribution of Gal-LNC 5 reached the liver majorly (Figure 6c). LNC is localized to all organs, including the liver, spleen, lungs, heart, and kidney in BALB/c mice. The control group (not injected anything) did not show any fluorescence. It released that there is no autofluorescence of the organ. The epifluorescence of the liver reached around 1.24 × 10^10^, whereas other organs are 8.7 × 10^8^. The fold decreases for the spleen (21.44092219), lungs (14.1337386), heart (30.46683047), and kidney (12.08576998) compared to the liver without affecting the nature of organs. This Gal-LNC5 could be an excellent vector to deliver nucleic acids for liver disorders.

## 4. Discussion

This study demonstrates the successful development of a novel galactosylated lipid nanoparticle (Gal-LNC 5) system for efficient liver-specific delivery of gain of function variants, Padua FIX-L plasmid, to hepatic cell lines and liver. The Gal-LNC exhibits optimal physicochemical properties, including hydrodynamic diameters within 200 nm, uniform dispersion in solution with low polydispersity index (PDI < 0.5), and a positive surface charge of less than +70 mV. These characteristics are the reason for strong nucleic acid binding and facilitate the nanoparticles for efficient intracellular delivery, making them promising candidates for non-viral gene therapy applications. The gel retardation assay confirms robust complexation of all Gal-LNC variants with plasmid DNA (pDNA) across various lipid-to-nucleic acid ratios, varying the lipid ratio from 1:1, 2:1, 4:1, and 8:1. The heparin displacement assay further confirms the stability of lipoplexes that they can be stable in competitive interactions with negatively charged molecules in the systemic settings, which is crucial for maintaining the integrity of the pDNA cargo during in vivo delivery. The in vitro transfection experiments show the superior transfection efficiency of the Gal-LNC 5 than LF in HepG2 cell lines. The enhanced gene expression observed with the Gal-LNC 5 can be attributed to their ability to target the asialoglycoprotein receptors (ASGPRs), which are highly expressed on the surface of hepatocytes, facilitating receptor-mediated endocytosis and improved intracellular delivery of lipid nanoparticles. The cloning and expression of the Padua variant of factor IX (FIX-L) in a CpG-free backbone further optimizes the therapeutic potential of a lipid nanoparticle-enabled gene therapy approach. The Padua variant exhibits an 8-fold increase in clotting activity compared to the wild-type FIX. At the same time, the removal of CpG dinucleotides further enhances the degree and duration of transgene expression in hepatic cell lines. The Western blot analysis confirms the production of FIX protein by the Gal-LNC 5-mediated delivery of the FIX-L plasmid in both HEK293T and HepG2 cell lines. More importantly, the intraperitoneal administration of Gal-LNC 5 shows more specific biodistribution in the liver than non-targeted LNC.

### Limitations of the Study

In vivo therapeutic evaluation of the gene therapy strategy with FIX pDNA-encapsulated Gal-LNC in a transgenic animal model with factor IX knock-out (hemophilia B mouse model) must be performed to complete the pre-clinical evaluations.

## 5. Conclusions

In conclusion, this study presents a promising strategy for treating hemophilia B using a liver-specific lipid nanoparticle-based gene therapy approach. The developed Gal-LNC system efficiently delivers a CpG-free Padua FIX plasmid to hepatic cell lines, producing functional FIX protein at a therapeutically relevant level. The optimal physicochemical properties, enhanced transfection efficiency, serum stability, and liver-specific delivery make the Gal-LNC a promising candidate for further in vivo evaluation and potential clinical translation. Optimization of the liver-targeting lipid nanoparticle system and the use of the hyperactive Padua FIX variant in a CpG-free backbone pave the way for the development of novel lipid nanoparticle-based gene therapy products for hemophilia B and multiple other monogenic liver disorders.

## Figures and Tables

**Table 1:** Composition of individual lipids in different liposomal formulations.

Liposome	16 Cy-6-Gal (mM)	Amide (mM)	DOPE (mM)	Chol (mM)
Gal-LNC 1	0.25	1	1	1
Gal-LNC 2	0.25	1	0.5	1
Gal-LNC 3	0.25	1	0.25	1
Gal-LNC 4	0.25	1	0	1
Gal-LNC 5	0.25	1	1	0.5
Gal-LNC 6	0.25	1	1	0.25
Gal-LNC 7	0.25	1	1	0
Gal-LNC 8	0.25	1	0.5	0.5
Gal-LNC 9	0.25	1	0.5	0.25
Gal-LNC 10	0.25	1	0.5	0
Gal-LNC 11	0.25	1	0.25	0.5
Gal-LNC 12	0.25	1	0	0.5
LNC	-	1	1	0.25

## Data Availability

All raw data are included in the Supplementary Materials.

## Supplementary Materials

The following supporting information can be downloaded at: https://www.mdpi.com/article/10.3390/pharmaceutics16111427/s1. Gel images and statistical data are provided in the supplementary information.

## References

[1] Iorio A., Stonebraker J.S., Chambost H., Makris M., Coffin D., Herr C., Germini F. (2019). Establishing the Prevalence and Prevalence at Birth of Hemophilia in Males: A Meta-analytic Approach Using National Registries. Ann. Intern. Med..

[2] Stonebraker J.S., Bolton-Maggs P.H., Soucie J.M., Walker I., Brooker M. (2010). A study of variations in the reported haemophilia A prevalence around the world. Haemoph. Off. J. World Fed. Hemoph..

[3] Knobe K., Berntorp E. (2011). Haemophilia and joint disease: Pathophysiology, evaluation, and management. J. Comorbidity.

[4] Peyvandi F., Garagiola I., Young G. (2016). The past and future of haemophilia: Diagnosis, treatments, and its complications. Lancet.

[5] Armenteros-Yeguas V., Gárate-Echenique L., Tomás-López M.A., Cristóbal-Domínguez E., Moreno-de Gusmão B., Miranda-Serrano E., Moraza-Dulanto M.I. (2017). Prevalence of difficult venous access and associated risk factors in highly complex hospitalised patients. J. Clin. Nurs..

[6] Srivastava A. (2004). Dose and response in haemophilia—Optimization of factor replacement therapy. Br. J. Haematol..

[7] Srivastava A., You S.K., Ayob Y., Chuansumrit A., de Bosch N., Perez Bianco R., Ala F. (2005). Hemophilia treatment in developing countries: Products and protocols. Semin. Thromb. Hemost..

[8] Mathews V., Viswabandya A., Baidya S., George B., Nair S., Chandy M., Srivastava A. (2005). Surgery for hemophilia in developing countries. Semin. Thromb. Hemost..

[9] Cancio M.I., Reiss U.M., Nathwani A.C., Davidoff A.M., Gray J.T. (2013). Developments in the treatment of hemophilia B: Focus on emerging gene therapy. Appl. Clin. Genet..

[10] Rogers G.L., Herzog R.W. (2015). Gene therapy for hemophilia. Front. Biosci..

[11] Soroka A.B., Feoktistova S.G., Mityaeva O.N., Volchkov P.Y. (2023). Gene Therapy Approaches for the Treatment of Hemophilia B. Int. J. Mol. Sci..

[12] Nathwani A.C. (2019). Gene therapy for hemophilia. Hematol. Am. Soc. Hematol. Educ. Program.

[13] VandenDriessche T., Chuah M.K. (2018). Hyperactive Factor IX Padua: A Game-Changer for Hemophilia Gene Therapy. Mol. Ther. J. Am. Soc. Gene Ther..

[14] Mendell J.R., Connolly A.M., Lehman K.J., Griffin D.A., Khan S.Z., Dharia S.D., Quintana-Gallardo L., Rodino-Klapac L.R. (2022). Testing preexisting antibodies prior to AAV gene transfer therapy: Rationale, lessons and future considerations. Mol. Ther. Methods Clin. Dev..

[15] Louis Jeune V., Joergensen J.A., Hajjar R.J., Weber T. (2013). Pre-existing anti-adeno-associated virus antibodies as a challenge in AAV gene therapy. Hum. Gene Ther. Methods.

[16] Klamroth R., Hayes G., Andreeva T., Gregg K., Suzuki T., Mitha I.H., Hardesty B., Shima M., Pollock T., Slev P. (2022). Global Seroprevalence of Pre-existing Immunity Against AAV5 and Other AAV Serotypes in People with Hemophilia A. Hum. Gene Ther..

[17] High K.A., Anguela X.M. (2015). Adeno-associated viral vectors for the treatment of hemophilia. Hum. Mol. Genet..

[18] Brimble M.A., Reiss U.M., Nathwani A.C., Davidoff A.M. (2016). New and improved AAVenues: Current status of hemophilia B gene therapy. Expert Opin. Biol. Ther..

[19] Monahan P.E., Négrier C., Tarantino M., Valentino L.A., Mingozzi F. (2021). Emerging Immunogenicity and Genotoxicity Considerations of Adeno-Associated Virus Vector Gene Therapy for Hemophilia. J. Clin. Med..

[20] Ertl H.C.J. (2022). Immunogenicity and toxicity of AAV gene therapy. Front. Immunol..

[21] Fu Q., Polanco A., Lee Y.S., Yoon S. (2023). Critical challenges and advances in recombinant adeno-associated virus (rAAV) biomanufacturing. Biotechnol. Bioeng..

[22] Alton E., Armstrong D.K., Ashby D., Bayfield K.J., Bilton D., Bloomfield E.V., Boyd A.C., Brand J., Buchan R., Calcedo R. (2015). Repeated nebulisation of non-viral CFTR gene therapy in patients with cystic fibrosis: A randomised, double-blind, placebo-controlled, phase 2b trial. Lancet Respir. Med..

[23] Coelho T., Adams D., Silva A., Lozeron P., Hawkins P.N., Mant T., Perez J., Chiesa J., Warrington S., Tranter E. (2013). Safety and efficacy of RNAi therapy for transthyretin amyloidosis. N. Engl. J. Med..

[24] Charbon V., Latour I., Lambert D.M., Buc-Calderon P., Neuvens L., De Keyser J.L., Gallez B. (1996). Targeting of drug to the hepatocytes by fatty acids. Influence of the carrier (albumin or galactosylated albumin) on the fate of the fatty acids and their analogs. Pharm. Res..

[25] Merwin J.R., Noell G.S., Thomas W.L., Chiou H.C., DeRome M.E., McKee T.D., Spitalny G.L., Findeis M.A. (1994). Targeted delivery of DNA using YEE(GalNAcAH)3, a synthetic glycopeptide ligand for the asialoglycoprotein receptor. Bioconjugate Chem..

[26] Mamidyala S.K., Dutta S., Chrunyk B.A., Préville C., Wang H., Withka J.M., McColl A., Subashi T.A., Hawrylik S.J., Griffor M.C. (2012). Glycomimetic ligands for the human asialoglycoprotein receptor. J. Am. Chem. Soc..

[27] Arjunan P., Kathirvelu D., Mahalingam G., Goel A.K., Zacharaiah U.G., Srivastava A., Marepally S. (2024). Lipid-nanoparticle-enabled nucleic acid therapeutics for liver disorders. Acta Pharm. Sin. B.

[28] Mukthavaram R., Marepally S., Venkata M.Y., Vegi G.N., Sistla R., Chaudhuri A. (2009). Cationic glycolipids with cyclic and open galactose head groups for the selective targeting of genes to mouse liver. Biomaterials.

[29] Samelson-Jones B.J. (2022). Worldwide use of factor IX Padua for hemophilia B gene therapy. Mol. Ther. J. Am. Soc. Gene Ther..

[30] Reyes-Sandoval A., Ertl H.C. (2004). CpG methylation of a plasmid vector results in extended transgene product expression by circumventing induction of immune responses. Mol. Ther. J. Am. Soc. Gene Ther..

[31] Mahalingam G., Rachamalla H.K., Arjunan P., Karuppusamy K.V., Periyasami Y., Mohan A., Subramaniyam K., Salma M., Rajendran V., Moorthy M. (2024). SMART-lipid nanoparticles enabled mRNA vaccine elicits cross-reactive humoral responses against the omicron sub-variants. Mol. Ther. J. Am. Soc. Gene Ther..

