# Supplementary material for: Lipid Nanoparticle-Mediated Liver-Specific Gene Therapy for Hemophilia B
Source: Pharmaceutics. 2024 Nov 9;16(11):1427. doi: 10.3390/pharmaceutics16111427 (PMC11597186; doi:10.3390/pharmaceutics16111427)
Supplement: Supplementary file 1 [file pharmaceutics-16-01427-s001.zip › pharmaceutics-3215118-supplementary.pdf]

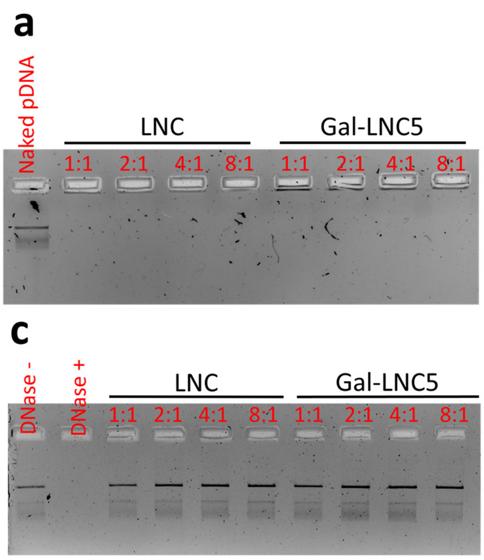

Figure S1. Liposomes complexation study of pDNA comparing LNC and Gal-LNC 5. (a) Electrophoretic mobility retardation assay of naked pDNA, LNC and Gal-LNC5 with charge ratios (1:1, 2:1, 4:1, and 8:1) (b) Heparin displacement assay of naked pDNA, LNC and Gal-LNC 5 lipoplex in the presence of heparin (c) DNase Sensitivity assay for LNC and Gal-LNC 5 in the presence of DNase enzyme.

**CpG Seq FP**	CTCAGTGGGCAGAGAGCACATG
**CpG Seq RP**	CACTGCATTCTAGTTGTGGTTTGTCC

Table S1: Primers for FIX-L cloning Confirmation

**hF9 gene FP**	GGCAAAGTGGACGCTTTCTG
**hF9 gene RP**	GGTCTCCTCGATGTTGTGCT

Table S2: qPCR primers

Comparison	Significance of GFP	P value
LF-MAX vs. Gal-LNC 1	****	<0.0001
LF-MAX vs. Gal-LNC 2	****	<0.0001
LF-MAX vs. Gal-LNC 3	****	<0.0001
LF-MAX vs. Gal-LNC 4	****	<0.0001
LF-MAX vs. Gal-LNC 5	ns	0.9998
LF-MAX vs. Gal-LNC 6	****	<0.0001
LF-MAX vs. Gal-LNC 7	****	<0.0001
LF-MAX vs. Gal-LNC 8	****	<0.0001
LF-MAX vs. Gal-LNC 9	****	<0.0001
LF-MAX vs. Gal-LNC 10	****	<0.0001
LF-MAX vs. Gal-LNC 11	****	<0.0001
LF-MAX vs. Gal-LNC 12	****	<0.0001
LF-MAX vs. LNC	****	<0.0001

Table S3: Statistical significance of LF compared with LNC and Gal-LNC 5 in Hek 293T

Comparison	Significance of GFP	P value
LF-MAX vs. Gal-LNC 1	****	<0.0001
LF-MAX vs. Gal-LNC 2	****	<0.0001
LF-MAX vs. Gal-LNC 3	****	<0.0001
LF-MAX vs. Gal-LNC 4	****	<0.0001
LF-MAX vs. Gal-LNC 5	ns	0.0634
LF-MAX vs. Gal-LNC 6	****	<0.0001
LF-MAX vs. Gal-LNC 7	****	<0.0001
LF-MAX vs. Gal-LNC 8	****	<0.0001
LF-MAX vs. Gal-LNC 9	****	<0.0001
LF-MAX vs. Gal-LNC 10	****	<0.0001
LF-MAX vs. Gal-LNC 11	****	<0.0001
LF-MAX vs. Gal-LNC 12	****	<0.0001
LF-MAX vs. LNC	ns	0.5078

Table S4: Statistical significance of LF compared with LNC and Gal-LNC 5 in HepG2
